# Primary Role of the Kidney in Pathogenesis of Hypertension

**DOI:** 10.3390/life14010119

**Published:** 2024-01-14

**Authors:** Gheun-Ho Kim

**Affiliations:** Department of Internal Medicine, Hanyang University College of Medicine, Seoul 04763, Republic of Korea; kimgh@hanyang.ac.kr; Tel.: +82-2-2290-8318

**Keywords:** inflammation, pressure-natriuresis, salt-sensitivity, sodium, vascular resistance

## Abstract

Previous transplantation studies and the concept of ‘nephron underdosing’ support the idea that the kidney plays a crucial role in the development of essential hypertension. This suggests that there are genetic factors in the kidney that can either elevate or decrease blood pressure. The kidney normally maintains arterial pressure within a narrow range by employing the mechanism of pressure-natriuresis. Hypertension is induced when the pressure-natriuresis mechanism fails due to both subtle and overt kidney abnormalities. The inheritance of hypertension is believed to be polygenic, and essential hypertension may result from a combination of genetic variants that code for renal tubular sodium transporters or proteins involved in regulatory pathways. The renin-angiotensin-aldosterone system (RAAS) and sympathetic nervous system (SNS) are the major regulators of renal sodium reabsorption. Hyperactivity of either the RAAS or SNS leads to a rightward shift in the pressure-natriuresis curve. In other words, hypertension is induced when the activity of RAAS and SNS is not suppressed despite increased salt intake. Sodium overload, caused by increased intake and/or reduced renal excretion, not only leads to an expansion of plasma volume but also to an increase in systemic vascular resistance. Endothelial dysfunction is caused by an increased intracellular Na^+^ concentration, which inhibits endothelial nitric oxide (NO) synthase and reduces NO production. The stiffness of vascular smooth muscle cells is increased by the accumulation of intracellular Na^+^ and subsequent elevation of cytoplasmic Ca^++^ concentration. In contrast to the hemodynamic effects of osmotically active Na^+^, osmotically inactive Na^+^ stimulates immune cells and produces proinflammatory cytokines, which contribute to hypertension. When this occurs in the gut, the microbiota may become imbalanced, leading to intestinal inflammation and systemic hypertension. In conclusion, the primary cause of hypertension is sodium overload resulting from kidney dysregulation.

## 1. Blood Pressure Goes with the Kidney

Blood pressure is a heritable trait because hypertension occurs with a familial tendency. The heritability of sitting and standing blood pressure was estimated to be between 39% and 63% in twins and between 16% and 22% in families [1]. Although the brain, kidneys, and blood vessels are major organs involved in regulating blood pressure and inducing hypertension, the kidneys have a unique relationship with blood pressure, suggesting that some genetic factors elevating or decreasing blood pressure exist in the kidney.

Transplantation studies, conducted on both experimental animals and humans, have provided evidence that “blood pressure goes with the kidney” [2]. When a kidney from a normotensive rat was transplanted into a young bilaterally nephrectomized hypertensive rat, the blood pressure of the hypertensive rat did not increase. Conversely, when a kidney from a young hypertensive rat (before the onset of hypertension) was transplanted into a bilaterally nephrectomized normotensive rat, the blood pressure of the normotensive rat increased [3]. Similarly, the high blood pressure of six patients with essential hypertension and terminal nephrosclerosis became normalized when they underwent bilateral nephrectomy and received a kidney transplant from a young normotensive donor. Thus, essential hypertension in humans is shown to be similar to the hypertension seen in spontaneously hypertensive rats in that both conditions can be corrected by kidney transplantation from a normotensive donor [4]. 

Numerous epidemiological studies suggest an inverse relationship between low birth weight and hypertension. A reduction in the number of nephrons, or nephron underdosing, has been proposed as a potential cause of essential hypertension [5]. This hypothesis was proven using three-dimensional stereology on autopsy kidneys, which revealed a lower number of glomeruli per kidney in patients with hypertension compared to normotensive controls [6]. Experiments using animal models of fetal programming further support this notion, indicating that programming during fetal life is a response to an adverse fetal environment. This process of programming leads to permanent adaptive responses, which in turn result in structural and physiological changes that ultimately contribute to the development of hypertension [7]. Accordingly, transplantation studies and the concept of ‘nephron underdosing’ support the idea that the kidney plays a crucial role in the development of essential hypertension.

## 2. Impaired Pressure-Natriuresis

What component of the kidney is critical for regulating blood pressure? The answer would be the kidneys’ control of urinary sodium excretion. Several decades ago, Dr. Guyton proposed ‘pressure-natriuresis’ by which blood pressure is tightly regulated within a narrow range through alterations in urinary sodium excretion [8]. The pressure-natriuresis mechanism is a feedback system that controls arterial pressure in the long term. It works by reducing sodium reabsorption and increasing sodium excretion in response to an increase in renal perfusion pressure. The specific intrarenal mechanism for the decrease in tubular reabsorption in response to increases in renal perfusion pressure appears to be related to hemodynamic factors such as medullary blood flow and renal interstitial hydrostatic pressure, as well as renal autocoids including nitric oxide, prostaglandins, kinins, and angiotensin II [9]. In addition to changes in renal Na^+^ transporters, alterations in tight junction proteins may be involved in the impairment of pressure-natriuresis [10].

**Figure 1:**
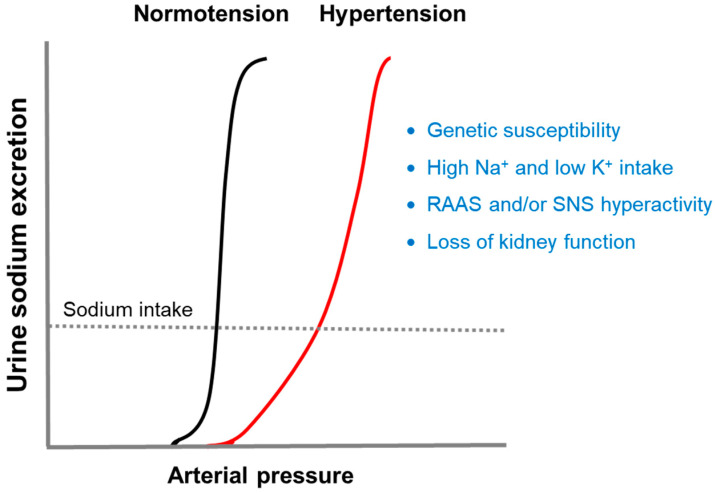
Pressure-natriuresis curves. The kidney normally maintains arterial pressure within a narrow range (normotension) by exerting the mechanism of pressure-natriuresis. If the normal pressure-natriuresis curve shifts to the right, hypertension will occur due to impaired renal sodium excretion. There are four major factors known to cause this change [11]. RAAS, renin-angiotensin-aldosterone system; SNS, sympathetic nervous system.

If the normal pressure-natriuresis curve shifts to the right, hypertension will occur due to impaired renal sodium excretion (Figure 1). Four major factors known to induce this alteration are genetic susceptibility, diet, neurohumoral activation, and loss of kidney function [11]. The following sections will discuss how these factors disrupt the pressure-natriuresis relationship.

### 2.1. Genetic Susceptibility and Salt Sensitivity

Primary or essential hypertension has a genetic basis, although we do not know which specific genes cause it [12]. The inheritance of hypertension is believed to be polygenic. Over the past decade, genome-wide association studies have identified more than 1000 single nucleotide polymorphisms that are associated with hypertension [13]. Individual single nucleotide polymorphisms have only a small effect on blood pressure. However, when multiple single nucleotide polymorphisms occur simultaneously, the risk of hypertension can be assessed using a polygenic risk score (PRS) [14]. The PRS is a reliable predictor of early-onset hypertension, with a progressive impact. Individuals who fall within the highest 2.5% of PRS have an almost threefold increased risk of developing hypertension, while a low PRS offers protection against it [15].

As a result of previous extensive genetic studies on human hypertension, some single-gene mutations were discovered that may either increase or decrease blood pressure. These components are present throughout the segments of the nephron and are directly or indirectly involved in the regulation of renal sodium transport. In particular, the genes are located in the distal nephron and code for tubular sodium transport systems or proteins that belong to regulatory pathways [16]. Hypertension is caused by gain-of-function mutations of the epithelial Na^+^ channel (ENaC) in Liddle syndrome. It is also caused by mutations in WNK4, WNK1, KLHL3, and CUL3, which increase the thiazide-sensitive Na^+^-Cl^−^ cotransporter (NCC) activity in pseudohypoaldosteronism type II. Additionally, hypertension can be caused by a chimeric 11 β-hydroxylase/aldosterone synthase gene in glucocorticoid-remediable aldosteronism. On the other hand, hypotension is induced by loss-of-function mutations of the NCC in Gitelman syndrome, as well as loss-of-function mutations of Na^+^-K^+^-2Cl^−^ cotransporter 2 (NKCC2), the renal outer medullary potassium channel (ROMK), or the ClC-Kb chloride channel in Bartter syndrome. Although monogenic hypertension is very rare, these single-gene mutations have provided insight into the role of renal sodium handling in the development of hypertension.

Salt sensitivity of blood pressure is characterized by an increase in blood pressure following salt loading or a decrease in blood pressure following salt depletion. It is a trait observed in both humans and animals [17], and it can be identified in half of the hypertensive population and one-fourth of normotensive subjects [18]. We believe that salt sensitivity can be attributed to genetic susceptibility. According to Manosroi and Williams [19], 18 genes were found to be associated with salt-sensitive hypertension (Table 1). These genes may account for approximately 50% of the population with primary hypertension. However, only eight genes were found to be associated with lower renin levels. Validation and further characterizations are necessary.

From the lessons learned from monogenic hypertension, it is conceivable that salt sensitivity is caused by a combination of genetic variants that code for tubular sodium transporters or proteins involved in regulatory pathways. Whereas a single nucleotide polymorphism has no significant effect on salt sensitivity, the interaction of multiple combined single nucleotide polymorphisms can lead to salt-sensitive hypertension. Manunta et al. evaluated the separate and combined impacts of the ADD1 (Gly460Trp), WNK1 (rs880054 A/G), and NEDD4L (rs4149601 G/A) polymorphisms on the renal and blood pressure responses to an acute salt load, the reduction in blood pressure after one month of treatment with 12.5 mg of hydrochlorothiazide, and ambulatory 24-h blood pressure. Individually, the variants exhibited modest effects on the specific phenotypes that were studied. However, they found that relatively common alleles in the ADD1, WNK1, and NEDD4L genes, when present in combination, have significant effects on renal sodium handling, blood pressure, and antihypertensive response to thiazides [20].

### 2.2. Sodium and Potassium Intake 

It is well known that a high sodium intake is associated with elevated blood pressure. In multiple populations, the rise in blood pressure with age is directly correlated with increasing levels of sodium intake. Multiple scattered groups who consume less than 50 mmol of sodium per day have little or no hypertension. When individuals consume excessive amounts of sodium, hypertension can develop. [21]. Sodium restriction to a level below 100 mmol per day will lower blood pressure in most individuals [22].

Sodium taken into the body is primarily localized in the extracellular space due to the activity of Na^+^/K^+^-ATPase, which is present in all cell membranes. To maintain the osmolality of extracellular fluid and cell volumes, sodium intake is typically accompanied by water retention. The resulting expansion of extracellular fluid and plasma volume can lead to hypertension if the mechanism of pressure-natriuresis fails to work. However, a certain portion of sodium is not confined to the extracellular space. After an increase in dietary salt, the excess sodium accumulates in the subcutaneous interstitium, binding to proteoglycans. This osmotically inactive Na^+^ storage contributes to regulating body sodium balance and blood pressure, as it can be drained through the lymphatics [23]. The increased interstitial tonicity activates the tonicity-responsive enhancer binding protein (TonEBP) in macrophages that infiltrate the interstitium of the skin. TonEBP transactivates the vascular endothelial growth factor C (VEGF-C) gene and enhances VEGF-C secretion by macrophages. This increases lymphatic capillary density and attenuates the blood pressure response to high salt [24]. 

Epidemiological studies suggest an association between potassium intake and blood pressure. A previous meta-analysis of 22 randomized controlled trials and 11 cohort studies has shown that increased potassium intake reduces blood pressure in individuals with hypertension [25]. In a study involving 102,216 adults from 18 countries, researchers found an inverse relationship between urinary potassium excretion and systolic blood pressure. This relationship was more pronounced in individuals with hypertension compared to those without it, and it became steeper with increasing age [26]. The analysis of the Dietary Approaches to Stop Hypertension Sodium Trial (DASH-Sodium) dataset showed that systolic blood pressure increased when potassium intake was less than 1 g per day [27].

Potassium deficiency can be linked to the development of salt-sensitive hypertension because it reduces the secretion of K^+^ while increasing the retention of Na^+^ [28]. Low dietary K^+^ intake activates NCC in the distal convoluted tubule, leading to an increase in NaCl reabsorption. The resulting low distal Na^+^ delivery will reduce K^+^ secretion through ROMK in the connecting tubule and cortical collecting duct. A high K^+^ diet, on the contrary, may relieve hypertension by inducing natriuresis (NCC downregulation) and direct vascular effects for vasodilation and decalcification [29].

### 2.3. Renin-Angiotensin-Aldosterone System (RAAS)

The RAAS is one of the major regulators of renal sodium reabsorption. Renin is secreted from the kidney and cleaves angiotensinogen, which is produced by the liver, into angiotensin I. Angiotensin I is converted to angiotensin II by the enzyme angiotensin-converting enzyme (ACE), which is found in the lungs and kidneys.

Angiotensin II induces hypertension by increasing renal sodium reabsorption and constricting arterioles. Hypertension is induced when angiotensin II is not suppressed despite increased salt intake. Rats with angiotensin II-induced hypertension exhibit a rightward shift in the pressure-natriuresis curve, primarily due to a significant impairment of sodium excretion. The reversal of these effects by losartan suggests that the AT1 receptor mediates the shift in the pressure-natriuresis curve in angiotensin II-induced hypertension [30,31].

There is local and independent control of angiotensin II within the kidney, which influences sodium excretion and regulates blood pressure. Angiotensin II is formed intrarenally from systemically delivered angiotensin I and intrarenally formed angiotensin I through ACE. Proximal tubule cells are believed to secrete angiotensin II into the tubular fluid in order to activate luminal angiotensin II receptors [32]. The AT1 receptors are located on the apical and basal membranes of the proximal tubule, as well as on the basal membrane of collecting duct cells. In the proximal tubule, angiotensin II binds to the AT1 receptor and upregulates the Na^+^/H^+^ exchanger 3 (NHE3). In animal models, NHE3 plays a critical role in the development of angiotensin II-induced hypertension [33]. Moreover, the genetic deletion of NHE3 specifically in the proximal tubules of the kidney reduces blood pressure by enhancing the pressure natriuretic response [34].

In the collecting duct’s principal cells, angiotensin II binds to the AT1 receptor and upregulates the ENaC. In the collecting duct’s type B intercalated cells, angiotensin II induces dephosphorylation of the mineralocorticoid receptor (MR) through the AT1 receptor. This renders the MR susceptible to aldosterone binding, which stimulates pendrin [35]. Pendrin is a Cl^−^/HCO_3_^−^ exchanger that regulates extracellular fluid volume and electrolyte balance downstream of aldosterone signaling [36].

Angiotensin II also acts on the adrenal glands to produce aldosterone, which promotes sodium reabsorption and induces hypertension. The NCC and ENaC are the major sodium transporters regulated by aldosterone in the distal convoluted tubule and collecting duct, respectively. Serum- and glucocorticoid-regulated kinase 1 (SGK1) is a crucial gene product induced by aldosterone in the distal nephron [37]. The SGK1-Nedd4-2 pathway affects the trafficking of ENaC and the abundance of NCC protein [38].

In addition to the kidney, MR is present in virtually all cells of the cardiovascular system, including endothelial cells, vascular smooth muscle cells (VSMCs), and cardiomyocytes. Increased signaling of endothelial cell MR can lead to the activation of endothelial sodium channels, reduced production of nitric oxide, oxidative stress, and inflammation [39]. MR activation in VSMCs directly contributes to vascular oxidative stress, vasoconstriction, and arterial hypertension [40]. MR signaling in innate and adaptive immune cells can also contribute to hypertension and hypertensive end-organ damage by upregulating the expression of proinflammatory genes [41]. 

### 2.4. Sympathetic Nervous System (SNS)

The SNS activity is another crucial factor in regulating blood pressure. Activation of renal sympathetic (efferent) nerves increases tubular sodium reabsorption, renin release, and renal vascular resistance. This, in turn, leads to a shift of the pressure-natriuresis curve to the right and contributes to the chronic elevation of blood pressure [42]. Consistent with this concept, rats with experimental and spontaneous hypertension undergo a significant reduction in blood pressure after renal denervation [43]. Renal denervation may be an adjunct treatment option in uncontrolled resistant hypertension. It may be considered as an alternative for patients who are unable to tolerate long-term medications at the required doses or cannot tolerate medications at all [44].

Central mechanisms in the brainstem and hypothalamus can modulate the level of renal sympathetic nerve activity. The rostral ventrolateral medulla (RVLM) is responsible for the basal and reflex control of sympathetic activity. RVLM neurons project to sympathetic preganglionic neurons in the spinal cord, which then project to the kidneys via postganglionic neurons. These postganglionic neurons innervate the three major neuroeffectors in the kidney [45]. 

The actions of the SNS on the kidney are dependent on both α- and β-adrenergic receptors. Increased renal sympathetic nerve activity stimulates the secretion of renin by activating β_1_-adrenergic receptors in juxtaglomerular granular cells. It also results in an increase in renal tubular sodium reabsorption through the stimulation of α_1B_-adrenergic receptors in renal tubular epithelial cells. Additionally, it causes a decrease in renal blood flow through the stimulation of α_1A_-adrenergic receptors in the renal arterial resistance vessels [46].

The proximal tubule is the primary target of SNS activity due to its abundant innervation by sympathetic nerve fibers [47]. The α_1_-adrenergic receptors are mainly located on the basolateral membrane of the proximal tubule cell. It has been demonstrated that in primary cultures of proximal tubular cells, the abundance and activity of brush-border NHE3 are stimulated by norepinephrine and completely prevented by prior exposure to prazosin [48]. Therefore, the SNS upregulates NHE3 in the proximal tubule through an α_1_-adrenoceptor-mediated mechanism. Intrarenal angiotensin II activity may also be stimulated by SNS hyperactivity in the proximal tubule [49].

The increased SNS activity in the kidney induces NCC activation, resulting in sodium retention and salt-sensitive hypertension [50]. Mouse distal convoluted tubule cells are enriched with β_1_-adrenergic receptors, and norepinephrine rapidly increases the abundance of phosphorylated NCC, at least partially through oxidative stress-response kinase 1 (OSR1) [51]. In patients with essential hypertension, β-adrenergic receptor blockade increases plasma atrial natriuretic peptide levels, leading to improved pressure-natriuresis [52].

The kidney is also innervated by sensory (afferent) nerve fibers, which transmit information to the brain to modulate sympathetic outflow. Renal afferent neurons have small- to medium-sized cell bodies located in the lower thoracic and upper lumbar dorsal root ganglia and terminate in the spinal cord and the brainstem. Sensory nerve fibers are associated with all branches of the renal arteries in varying densities, and the renal pelvis has the highest density of sensory innervation compared to other structural components of the kidney. Renal sensory nerve fibers are typically categorized as mechanoreceptors and chemoreceptors. Mechanoreceptors in the renal pelvis detect changes in pelvic pressure caused by the flow of urine. Type R1 chemoreceptors respond to renal ischemia, whereas R2 chemoreceptors sense changes in ionic composition [53].

The SNS mediates short-term increases in blood pressure, and heart rate may serve as a marker of sympathetic activity [54]. However, the evidence regarding whether psychosocial stress leads to chronic hypertension is mixed [55]. In a multicenter longitudinal study of 4762 young adults initially aged 18 to 30 years, the authors found that heart rate was an independent predictor of future diastolic hypertension [56]. Therefore, beta-blockers can be considered the first-line treatment for diastolic hypertension in young adults.

### 2.5. Loss of Kidney Function

With a decrease in glomerular filtration rate (GFR), the prevalence of salt sensitivity increases. A simultaneous decrease in sodium excretion will ultimately increase blood volume and cardiac output, thereby elevating blood pressure to the set point. Therefore, salt sensitivity reflects a failure of the kidneys to excrete sufficient salt in response to an increase in salt intake due to an underlying defect in the pressure-natriuresis response [57].

The fact that reducing the functioning renal mass induces salt-sensitive hypertension is known from the early animal experiments conducted by Guyton and his associates. In dogs with 70% of their total renal tissue removed, arterial pressure increased by 30% to 40% within 48 to 72 h after drinking isotonic saline. The elevated blood pressure was reduced to normal levels within 24 h by simply allowing the dogs to drink tap water again [58]. 

A subtle kidney injury without an overt decline in GFR may cause salt sensitivity [59]. Several factors were known to contribute to the high prevalence of salt-sensitive hypertension in patients with chronic kidney disease (CKD). First, CKD is associated with the inappropriate activation of the RAAS, leading to the accumulation of angiotensin II in the body. Second, renal injury increases sympathetic tone, even when the GFR remains unchanged. This results from stimulating afferent signals originating from the kidney [42]. Following renal injury, there are immediate increases in catecholamine turnover in the brain, accompanied by rises in blood pressure and renal sympathetic nerve activity [60]. Overactivity of the SNS in CKD stimulates renin production by the renal juxtaglomerular cells. Additionally, increased levels of angiotensin II in patients with CKD can directly stimulate SNS activity. Third, vascular endothelial dysfunction is associated with kidney dysfunction. CKD is a condition characterized by increased oxidative stress, impaired nitric oxide (NO) production, and elevated endothelin levels [61]. Moreover, renal sodium transporters may exhibit inadequate responses to salt intake in damaged kidneys.

## 3. Sodium and Vascular Resistance

Sodium overload, caused by increased intake and/or reduced renal excretion, not only leads to an expansion of plasma volume but also to an increase in systemic vascular resistance. Guyton and his associates have shown that salt loading in a partially nephrectomized dog causes elevated arterial pressure [62]. This increase in pressure is initially caused by an increase in cardiac output, but it is eventually sustained by an increase in peripheral vascular resistance. Similarly, diuretic-induced salt depletion decreases vascular resistance. The initial drop in blood pressure after the administration of a diuretic is caused by a decrease in extracellular fluid and plasma volume. After prolonged use of diuretics, lower blood pressure is mostly related to decreased peripheral vascular resistance [63].

Salt-sensitive hypertension may be explained by an increase in total peripheral vascular resistance, rather than a change in cardiac output [57]. In approximately half of the subjects who are salt-resistant, the salt load is accommodated and eventually excreted without causing a significant increase in blood pressure. Salt-sensitive subjects, on the other hand, exhibit an abnormal vascular response. Vasodilation induced by dietary salt is diminished, peripheral resistance does not decrease, and salt intake leads to a significant increase in blood pressure [64]. The activation of RAAS and SNS both contribute to the failure of the peripheral vasculature to dilate rapidly, resulting in salt sensitivity.

How does an increase in sodium load affect peripheral vascular resistance and blood pressure? Ouabain, a selective Na^+^/K^+^-ATPase α2 inhibitor, is synthesized in the adrenal cortex and released into the plasma. Its plasma level increases in response to plasma volume expansion. In VSMCs, the cytoplasmic sodium concentration is increased by the action of ouabain, resulting in the reverse activation of Na^+^/Ca^++^ exchanger 1 (NCX1). The resulting increase in cytoplasmic calcium concentration stimulates excitation-contraction coupling, which leads to an increase in vascular tone and contractility. This vasotonic effect can be linked to hypertension [65]. Calcium channel blockers inhibit the influx of Ca^2+^ into vascular smooth muscle, leading to a decrease in vascular resistance [66].

**Figure 2:**
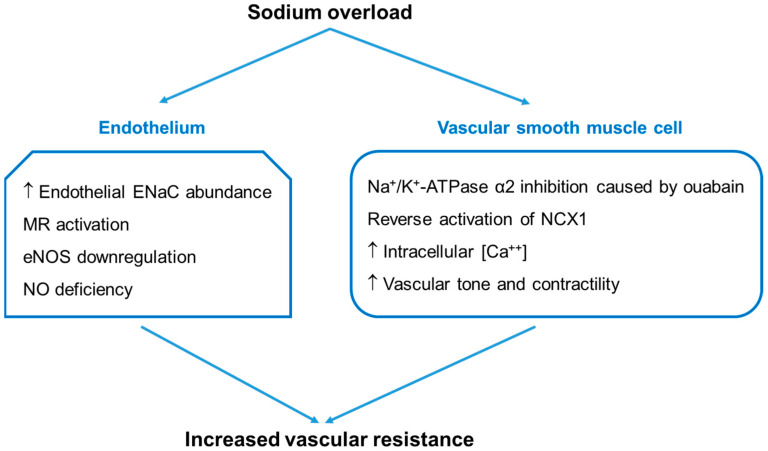
Sodium overload and increased vascular resistance. Sodium overload can affect both the endothelium and vascular smooth muscle cells, resulting in an increase in systemic vascular resistance. Endothelial dysfunction occurs when the intracellular Na^+^ concentration rises due to the activation of endothelial ENaC and MR, leading to the inhibition of eNOS and a decrease in NO production. Vascular smooth muscle stiffness is increased by the accumulation of intracellular Na^+^ (caused by ouabain) and subsequent elevation of cytoplasmic Ca^++^ concentration. ENaC, epithelial sodium channel; eNOS, endothelial nitric oxide synthase; MR, mineralocorticoid receptor; NCX1, Na^+^/Ca^++^ exchanger 1; NO, nitric oxide.

Endothelial function is also affected by sodium overload. High salt intake or elevated aldosterone levels can lead to impaired NO production by endothelial cells [67]. Endothelial ENaC is activated in response to the increased plasma sodium concentration, which enhances the transport of sodium into the cells. The subsequent increase in intracellular sodium concentration inhibits endothelial NO synthase (eNOS) and reduces NO production, resulting in vascular dysfunction [68]. Therefore, sodium overload affects both the endothelium and VSMCs (Figure 2).

## 4. Sodium, Immunity, and Inflammation

Elevated concentrations of tissue or extracellular sodium activate immune cells, releasing proinflammatory cytokines, which can contribute to hypertension. Both the innate and adaptive immune systems are involved in the production of cytokines IL-6, TNF-α, and IL-17A in response to high sodium intake [69].

Monocytes/macrophages are the major cellular component of the innate immune system, which is characterized by rapid defense mechanisms against invading pathogens. High salt enters the monocyte/macrophage via NCX1, causing the upregulation of the nuclear factor of activated T cells 5 (NFAT5, also called TonEBP) and the subsequent production of NO by inducible NO synthase (NOS2). Finally, proinflammatory cytokines such as IL-1β, IL-6, and TNF-α are released in response to high salt [41].

Dendritic cells serve as a bridge between the innate and adaptive immune systems. They process foreign antigens and present the resulting antigenic peptides to T cells, activating them [41]. High salt enters the dendritic cell through ENaC. An increase in intracellular sodium concentration facilitates calcium influx via the NCX, leading to the activation of protein kinase C and NADPH oxidase. This, in turn, results in an increase in superoxide and protein modification by ketoaldehydes, known as isolevuglandin (isoLG)-protein adducts. These isoLGs incite an autoimmune-like reaction, leading to the release of proinflammatory cytokines interferon-γ, and IL-17, as well as an increase in blood pressure [68].

Exposure of B and T lymphocytes to a high extracellular sodium concentration also triggers a proinflammatory condition. High levels of salt enter the T cells through the NKCC1 transporter, which results in the upregulation of NFAT5 and its downstream target SGK1. Upregulation of the T-helper cell 17 (T_H_17) transcription marker RORγt mediates the transcription and translation of IL-23R, which is essential for T_H_17 induction and the secretion of IL-17A [41].

Pharmacological inhibition of B and T cells with the immunosuppressive drug mycophenolate mofetil (MMF) can also have antihypertensive effects in animals with salt-sensitive hypertension. MMF normalized blood pressure in spontaneously hypertensive rats [70] and Dahl salt-sensitive rats [71]. Similarly, MMF attenuated hypertension, kidney T-cell infiltration, and urinary IFN-γ excretion in rats treated with DOCA-salt [72]. The blood pressure-lowering effects of MMF and TNF-α blockade were demonstrated in patients with psoriasis or rheumatoid arthritis, although anti-inflammatory or immune suppressive therapy is not currently used in routine cases of hypertension [73].

## 5. Sodium, Microbiome, and Hypertension 

Along with its effects on immunity and inflammation, sodium may promote hypertension by altering the gut microbiota [14]. In healthy individuals, the gut microbiome is typically in a stable state of eubiosis and in relative equilibrium with the surrounding environment [74]. However, significant changes in the microbiome can occur in various disorders, including hypertension. This imbalanced microbiota, or dysbiosis, is characterized by a decrease in microbial diversity and an increase in proinflammatory species. A high-salt diet altered the gut microbiota and depleted *Lactobacillus murinus* in both mice and humans [75]. The reduction in *Lactobacillus murinus* led to a decrease in the production of indole-3-lactic acid, which was associated with the accumulation of proinflammatory T_H_17 cells. In addition, treatment with *Lactobacillus murinus* mitigated salt-sensitive hypertension by modulating T_H_17 cells [75].

*Bacteroides fragilis* is another important symbiont in the human gut, and it produces arachidonic acid as a metabolite. A high-salt diet reduced the levels of *Bacteroides fragilis* and arachidonic acid in the intestine. This reduction led to an increase in corticosterone production derived from the intestine, resulting in elevated levels of corticosterone in both the serum and intestine. As a result, blood pressure elevation was promoted [76].

Microbial metabolites can be directly delivered to conventional animals through gavage, ad libitum drinking water, or injections [77]. Adoptive transfer of fecal material from conventionally housed mice fed a high-salt diet to germ-free mice predisposed them to increased intestinal inflammation and hypertension. [78]. Therefore, sodium overload alters the microbiome, induces intestinal inflammation, and may contribute to systemic hypertension. A systematic review of randomized controlled trials investigating the role of probiotics on hypertension showed that *Lactobacillus*-containing probiotics were effective if used in sufficiently high doses and for at least 8 weeks [79].

## 6. Conclusions

**Figure 3:**
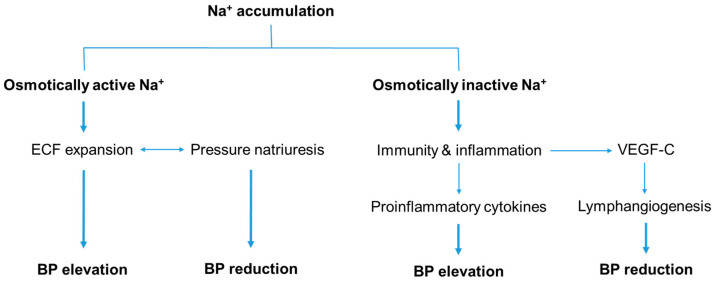
Sodium balance and blood pressure regulation. The sodium accumulated in the body can be divided into osmotically active and inactive Na^+^. Osmotically active Na^+^ is confined to the ECF and contributes to an increase in BP. Normally, pressure natriuresis helps to counteract hypertension. Osmotically inactive Na^+^ does not affect hemodynamics, but instead promotes inflammation, which contributes to hypertension. This result can be ameliorated by lymphangiogenesis, which is stimulated by the release of VEGF-C from the subcutaneous macrophages. BP, blood pressure; ECF, extracellular fluid; VEGF-C, vascular endothelial growth factor C.

By nature, the human kidney is programmed for sodium conservation. By contrast, habitual daily salt intake increases with age in Westernized countries. As individuals age, they are likely to experience a positive sodium balance, which can be linked to hypertension if the pressure-natriuresis mechanism does not function properly. The polygenic inheritance of essential hypertension can be explained by a combination of genetic variants that code for renal tubular sodium transporters or proteins involved in regulatory pathways. Abnormal activation of the RAAS and/or SNS also increases tubular sodium reabsorption, which leads to a rightward shift in the pressure-natriuresis curve. The salt sensitivity increases as the GFR declines. In addition to the hemodynamic effects of osmotically active Na^+^, the accumulation of osmotically inactive Na^+^ in the endothelium and VSMCs leads to an increase in vascular resistance. It also stimulates immune cells to release proinflammatory cytokines, contributing to hypertension. However, the osmotically inactive Na^+^ accumulated in the subcutaneous tissue can counteract hypertension through lymphangiogenesis (Figure 3). In conclusion, the primary cause of hypertension is sodium overload resulting from kidney dysregulation.

## Figures and Tables

**Table 1:** Genes reported to be associated with salt-sensitive hypertension.

Gene (SNP)	Molecule	Potential Mechanisms
ADRβ2 (rs1042713, rs1042714)	β2-Adrenergic receptor	Increased aldosterone secretion
LSD1 (rs587168, rs7548692)	Lysine-specific demethylase 1	Increased aldosterone secretion
CAV1 (rs3807989, rs926198, rs3840634)	Caveolin-1	Increased aldosterone secretion
STRN (rs2540923)	Striatin	Increased aldosterone secretion
CYP11B2 (rs1799998)	Aldosterone synthase	Increased aldosterone secretion
CYP17A1 (rs11191548, rs1004467, rs11191416)	17α-Hydroxylase	Increased aldosterone secretion
EDN1 (rs1800541, rs5370)	Endothelin-1	Increased aldosterone secretion
ESR2 (rs1256049, rs10144225)	Estrogen receptor 2	Increased aldosterone secretion
AGT (rs699, rs5050)	Angiotensinogen	Renal sodium retention
ADD-1 (rs4961)	Adducin-1	Renal sodium retention
SLC4A5 (rs7571842, rs10177833)	Electrogenic Na^+^/HCO_3_^−^ cotransporter 4	Renal sodium retention
STK39 (rs3754777, rs35929607, rs6749447)	Serine/threonine kinase 39	Renal sodium retention
WNK1 (rs11885, rs11554421 rs1468326)	Lysine-deficient protein kinase 1	Renal sodium retention
SGK1 (rs2758151, rs9402571, rs9376026)	Serum/Glucocorticoid regulated kinase 1	Adrenal and renal dysfunction
REN (rs6682082, rs6693954, rs5705)	Renin	Adrenal and renal dysfunction
ATP2B1 (rs2681472, rs17249754, rs2070759)	Plasma membrane Ca^++^ transporting ATPase 1	Vascular dysfunction
ACE (ACE I/D)	Angiotensin I converting enzyme 1	Renal and vascular dysfunction
BDKRB2 (rs11847625, rs334, I/D in exon 1)	Bradykinin receptor B2	Renal and vascular dysfunction

I/D, insertion/deletion; SNP, single nucleotide polymorphism. Data was collected from references [17,19].

## Data Availability

Not applicable.

## References

[1] Hunt S.C., Hasstedt S.J., Kuida H., Stults B.M., Hopkins P.N., Williams R.R. (1989). Genetic heritability and common environmental components of resting and stressed blood pressures, lipids, and body mass index in Utah pedigrees and twins. Am. J. Epidemiol..

[2] Adamczak M., Zeier M., Dikow R., Ritz E. (2002). Kidney and hypertension. Kidney Int. Suppl..

[3] Rettig R., Folberth C.G., Graf C., Kopf D., Stauss H., Unger T. (1991). Post-transplantation hypertension in recipients of renal grafts from hypertensive donor rats. Clin. Investig. Med..

[4] Curtis J.J., Luke R.G., Dustan H.P., Kashgarian M., Whelchel J.D., Jones P., Diethelm A.G. (1983). Remission of essential hypertension after renal transplantation. N. Engl. J. Med..

[5] Brenner B.M., Garcia D.L., Anderson S. (1988). Glomeruli and blood pressure. Less of one, more the other?. Am. J. Hypertens..

[6] Keller G., Zimmer G., Mall G., Ritz E., Amann K. (2003). Nephron number in patients with primary hypertension. N. Engl. J. Med..

[7] Alexander B.T. (2006). Fetal programming of hypertension. Am. J. Physiol. Regul. Integr. Comp. Physiol..

[8] Guyton A.C. (1991). Blood pressure control—Special role of the kidneys and body fluids. Science.

[9] Granger J.P., Alexander B.T., Llinas M. (2002). Mechanisms of pressure-natriuresis. Curr. Hypertens. Rep..

[10] Jo C.H., Kim S., Oh I.H., Park J.S., Kim G.H. (2017). Alteration of tight junction protein expression in Dahl salt-sensitive rat kidney. Kidney Blood Press. Res..

[11] Mizelle H.L., Montani J.P., Hester R.L., Didlake R.H., Hall J.E. (1993). Role of pressure-natriuresis in long-term control of renal electrolyte excretion. Hypertension.

[12] Carretero O.A., Oparil S. (2000). Essential hypertension. Part I: Definition and etiology. Circulation.

[13] Cabrera C.P., Ng F.L., Nicholls H.L., Gupta A., Barnes M.R., Munroe P.B., Caulfield M.J. (2019). Over 1000 genetic loci influencing blood pressure with multiple systems and tissues implicated. Hum. Mol. Genet..

[14] Harrison D.G., Coffman T.M., Wilcox C.S. (2021). Pathophysiology of Hypertension: The mosaic theory and beyond. Circ. Res..

[15] Vaura F., Kauko A., Suvila K., Havulinna A.S., Mars N., Salomaa V., FinnGen, Cheng S., Niiranen T. (2021). Polygenic risk scores predict hypertension onset and cardiovascular risk. Hypertension.

[16] Meneton P., Jeunemaitre X., de Wardener H.E., MacGregor G.A. (2005). Links between dietary salt intake, renal salt handling, blood pressure, and cardiovascular diseases. Physiol. Rev..

[17] Parksook W.W., Williams G.H. (2022). Challenges and approach to identifying individuals with salt sensitivity of blood pressure. Am. J. Nephrol..

[18] Weinberger M.H. (1991). Salt sensitivity as a predictor of hypertension. Am. J. Hypertens..

[19] Manosroi W., Williams G.H. (2019). Genetics of human primary hypertension: Focus on hormonal mechanisms. Endocr. Rev..

[20] Manunta P., Lavery G., Lanzani C., Braund P.S., Simonini M., Bodycote C., Zagato L., Delli Carpini S., Tantardini C., Brioni E. (2008). Physiological interaction between alpha-adducin and WNK1-NEDD4L pathways on sodium-related blood pressure regulation. Hypertension.

[21] Stamler J., Rose G., Stamler R., Elliott P., Dyer A., Marmot M. (1989). INTERSALT study findings. Public health and medical care implications. Hypertension.

[22] Sacks F.M., Svetkey L.P., Vollmer W.M., Appel L.J., Bray G.A., Harsha D., Obarzanek E., Conlin P.R., Miller E.R., Simons-Morton D.G. (2001). Effects on blood pressure of reduced dietary sodium and the Dietary Approaches to Stop Hypertension (DASH) diet. DASH-Sodium Collaborative Research Group. N. Engl. J. Med..

[23] Machnik A., Neuhofer W., Jantsch J., Dahlmann A., Tammela T., Machura K., Park J.K., Beck F.X., Müller D.N., Derer W. (2009). Macrophages regulate salt-dependent volume and blood pressure by a vascular endothelial growth factor-C-dependent buffering mechanism. Nat. Med..

[24] Marvar P.J., Gordon F.J., Harrison D.G. (2009). Blood pressure control: Salt gets under your skin. Nat. Med..

[25] Aburto N.J., Hanson S., Gutierrez H., Hooper L., Elliott P., Cappuccio F.P. (2013). Effect of increased potassium intake on cardiovascular risk factors and disease: Systematic review and meta-analyses. BMJ.

[26] Mente A., O’Donnell M.J., Rangarajan S., McQueen M.J., Poirier P., Wielgosz A., Morrison H., Li W., Wang X., Di C. (2014). Association of urinary sodium and potassium excretion with blood pressure. N. Engl. J. Med..

[27] Chaudhary P., Wainford R.D. (2021). Association of urinary sodium and potassium excretion with systolic blood pressure in the Dietary Approaches to Stop Hypertension Sodium Trial. J. Hum. Hypertens..

[28] Palmer B.F., Clegg D.J. (2020). Blood pressure lowering and potassium intake. J. Hum. Hypertens..

[29] Gritter M., Rotmans J.I., Hoorn E. (2019). Role of dietary K^+^ in natriuresis, blood pressure reduction, cardiovascular protection, and renoprotection. Hypertension.

[30] Van der Mark J., Kline R.L. (1994). Altered pressure-natriuresis in chronic angiotensin II hypertension in rats. Am. J. Physiol..

[31] Wang C.T., Chin S.Y., Navar L.G. (2000). Impairment of pressure-natriuresis and renal autoregulation in ANG II-infused hypertensive rats. Am. J. Physiol. Renal Physiol..

[32] Navar L.G., Harrison-Bernard L.M., Imig J.D., Wang C.T., Cervenka L., Mitchell K.D. (1999). Intrarenal angiotensin II generation and renal effects of AT1 receptor blockade. J. Am. Soc. Nephrol..

[33] Nwia S.M., Li X.C., Leite A.P.O., Hassan R., Zhuo J.L. (2022). The Na^+^/H^+^ exchanger 3 in the intestines and the proximal tubule of the kidney: Localization, physiological function, and key roles in angiotensin II-induced hypertension. Front. Physiol..

[34] Li X.C., Soleimani M., Zhu D., Rubera I., Tauc M., Zheng X., Zhang J., Chen X., Zhuo J.L. (2018). Proximal tubule-specific deletion of the NHE3 (Na^+^/H^+^ exchanger 3) promotes the pressure-natriuresis response and lowers blood pressure in mice. Hypertension.

[35] Coffman T.M. (2014). The inextricable role of the kidney in hypertension. J. Clin. Investig..

[36] Shibata S. (2019). Role of pendrin in the pathophysiology of aldosterone-induced hypertension. Am. J. Hypertens..

[37] McCormick J.A., Bhalla V., Pao A.C., Pearce D. (2005). SGK1: A rapid aldosterone-induced regulator of renal sodium reabsorption. Physiology.

[38] Arroyo J.P., Lagnaz D., Ronzaud C., Vázquez N., Ko B.S., Moddes L., Ruffieux-Daidié D., Hausel P., Koesters R., Yang B. (2011). Nedd4-2 modulates renal Na^+^-Cl^-^ cotransporter via the aldosterone-SGK1-Nedd4-2 pathway. J. Am. Soc. Nephrol..

[39] Jia G., Habibi J., Aroor A.R., Martinez-Lemus L.A., DeMarco V.G., Ramirez-Perez F.I., Sun Z., Hayden M.R., Meininger G.A., Mueller K.B. (2016). Endothelial mineralocorticoid receptor mediates diet-induced aortic stiffness in females. Circ. Res..

[40] McCurley A., Pires P.W., Bender S.B., Aronovitz M., Zhao M.J., Metzger D., Chambon P., Hill M.A., Dorrance A.M., Mendelsohn M.E. (2012). Direct regulation of blood pressure by smooth muscle cell mineralocorticoid receptors. Nat. Med..

[41] Hengel F.E., Benitah J.P., Wenzel U.O. (2022). Mosaic theory revised: Inflammation and salt play central roles in arterial hypertension. Cell. Mol. Immunol..

[42] Thomas P., Dasgupta I. (2015). The role of the kidney and the sympathetic nervous system in hypertension. Pediatr. Nephrol..

[43] Kline R.L., Stuart P.J., Mercer P.F. (1980). Effect of renal denervation on arterial pressure and renal norepinephrine concentration in Wistar-Kyoto and spontaneously hypertensive rats. Can. J. Physiol. Pharmacol..

[44] Barbato E., Azizi M., Schmieder R.E., Lauder L., Böhm M., Brouwers S., Bruno R.M., Dudek D., Kahan T., Kandzari D.E. (2023). Renal denervation in the management of hypertension in adults. A clinical consensus statement of the ESC Council on Hypertension and the European Association of Percutaneous Cardiovascular Interventions (EAPCI). Eur. Heart J..

[45] Díaz-Morales N., Baranda-Alonso E.M., Martínez-Salgado C., López-Hernández F.J. (2023). Renal sympathetic activity: A key modulator of pressure natriuresis in hypertension. Biochem. Pharmacol..

[46] DiBona G.F. (2005). Physiology in perspective: The Wisdom of the Body. Neural control of the kidney. Am. J. Physiol. Regul. Integr. Comp. Physiol..

[47] Barajas L., Liu L., Powers K. (1992). Anatomy of the renal innervation: Intrarenal aspects and ganglia of origin. Can. J. Physiol. Pharmacol..

[48] Healy V., Thompson C., Johns E.J. (2014). The adrenergic regulation of proximal tubular Na^+^/H^+^ exchanger 3 in the rat. Acta Physiol..

[49] Pontes R.B., Girardi A.C., Nishi E.E., Campos R.R., Bergamaschi C.T. (2015). Crosstalk between the renal sympathetic nerve and intrarenal angiotensin II modulates proximal tubular sodium reabsorption. Exp. Physiol..

[50] Kawarazaki W., Fujita T. (2021). Kidney and epigenetic mechanisms of salt-sensitive hypertension. Nat. Rev. Nephrol..

[51] Terker A.S., Yang C.L., McCormick J.A., Meermeier N.P., Rogers S.L., Grossmann S., Trompf K., Delpire E., Loffing J., Ellison D.H. (2014). Sympathetic stimulation of thiazide-sensitive sodium chloride cotransport in the generation of salt-sensitive hypertension. Hypertension.

[52] Nakaoka H., Kitahara Y., Amano M., Imataka K., Fujii J., Ishibashi M., Yamaji T. (1987). Effect of beta-adrenergic receptor blockade on atrial natriuretic peptide in essential hypertension. Hypertension.

[53] Osborn J.W., Tyshynsky R., Vulchanova L. (2021). Function of renal nerves in kidney physiology and pathophysiology. Annu. Rev. Physiol..

[54] Grassi G., Vailati S., Bertinieri G., Seravalle G., Stella M.L., Dell’Oro R., Mancia G. (1998). Heart rate as marker of sympathetic activity. J. Hypertens..

[55] Ziegler M.G., Milic M. (2017). Sympathetic nerves and hypertension in stress, sleep apnea, and caregiving. Curr. Opin. Nephrol. Hypertens..

[56] Kim J.R., Kiefe C., Liu K., Williams O.D., Jacobs D.R., Oberman A. (1999). Heart rate and subsequent blood pressure in young adults: The CARDIA study. Hypertension.

[57] Bailey M.A., Dhaun N. (2023). Salt sensitivity: Causes, consequences, and recent advances. Hypertension.

[58] Langston J.B., Guyton A.C., Douglas B.H., Dorsett P.E. (1963). Effect of changes in salt Intake on arterial pressure and renal function in partially nephrectomized dogs. Circ. Res..

[59] Johnson R.J., Herrera-Acosta J., Schreiner G.F., Rodriguez-Iturbe B. (2002). Subtle acquired renal injury as a mechanism of salt-sensitive hypertension. N. Engl. J. Med..

[60] Ye S., Ozgur B., Campese V.M. (1997). Renal afferent impulses, the posterior hypothalamus, and hypertension in rats with chronic renal failure. Kidney Int..

[61] Ku E., Lee B.J., Wei J., Weir M.R. (2019). Hypertension in CKD: Core Curriculum 2019. Am. J. Kidney Dis..

[62] Coleman T.G., Guyton A.C. (1969). Hypertension caused by salt loading in the dog. 3. Onset transients of cardiac output and other circulatory variables. Circ. Res..

[63] Danielson M. (1984). Hemodynamic effects of diuretic therapy in hypertension. Acta Pharmacol. Toxicol..

[64] Schmidlin O., Sebastian A.F., Morris R.C. (2007). What initiates the pressor effect of salt in salt-sensitive humans? Observations in normotensive blacks. Hypertension.

[65] Blaustein M.P., Zhang J., Chen L., Hamilton B.P. (2006). How does salt retention raise blood pressure?. Am. J. Physiol. Regul. Integr. Comp. Physiol..

[66] Sinha A.D., Agarwal R. (2019). Clinical pharmacology of antihypertensive therapy for the treatment of hypertension in CKD. Clin. J. Am. Soc. Nephrol..

[67] Fels J., Oberleithner H., Kusche-Vihrog K. (2010). Ménage à trois: Aldosterone, sodium and nitric oxide in vascular endothelium. Biochim. Biophys. Acta.

[68] Mutchler S.M., Kirabo A., Kleyman T.R. (2021). Epithelial sodium channel and salt-sensitive hypertension. Hypertension.

[69] Ertuglu L.A., Kirabo A. (2022). Dendritic cell epithelial sodium channel in inflammation, salt-sensitive hypertension, and kidney damage. Kidney360.

[70] Rodríguez-Iturbe B., Quiroz Y., Nava M., Bonet L., Chávez M., Herrera-Acosta J., Johnson R.J., Pons H.A. (2002). Reduction of renal immune cell infiltration results in blood pressure control in genetically hypertensive rats. Am. J. Physiol. Renal Physiol..

[71] Mattson D.L., James L., Berdan E.A., Meister C.J. (2006). Immune suppression attenuates hypertension and renal disease in the Dahl salt-sensitive rat. Hypertension.

[72] Moes A.D., Severs D., Verdonk K., van der Lubbe N., Zietse R., Danser A.H.J., Hoorn E.J. (2018). Mycophenolate mofetil attenuates DOCA-salt hypertension: Effects on vascular tone. Front. Physiol..

[73] Van Beusecum J.P., Moreno H., Harrison D.G. (2022). Innate immunity and clinical hypertension. J. Hum. Hypertens..

[74] Human Microbiome Project Consortium (2012). Structure, function and diversity of the healthy human microbiome. Nature.

[75] Wilck N., Matus M.G., Kearney S.M., Olesen S.W., Forslund K., Bartolomaeus H., Haase S., Mähler A., Balogh A., Markó L. (2017). Salt-responsive gut commensal modulates T_H_17 axis and disease. Salt-responsive gut commensal modulates T_H_17 axis and disease. Nature.

[76] Yan X., Jin J., Su X., Yin X., Gao J., Wang X., Zhang S., Bu P., Wang M., Zhang Y. (2020). Intestinal flora modulates blood pressure by regulating the synthesis of intestinal-derived corticosterone in high salt-induced hypertension. Circ. Res..

[77] O’Donnell J.A., Zheng T., Meric G., Marques F.Z. (2023). The gut microbiome and hypertension. Nat. Rev. Nephrol..

[78] Ferguson J.F., Aden L.A., Barbaro N.R., Van Beusecum J.P., Xiao L., Simmons A.J., Warden C., Pasic L., Himmel L.E., Washington M.K. (2019). High dietary salt-induced dendritic cell activation underlies microbial dysbiosis-associated hypertension. JCI Insight.

[79] Khalesi S., Sun J., Buys N., Jayasinghe R. (2014). Effect of probiotics on blood pressure: A systematic review and meta-analysis of randomized, controlled trials. Hypertension.

